# A comparative study on performance of the conventional and fixed-bed membrane bioreactors for treatment of Naproxen from pharmaceutical wastewater

**DOI:** 10.1038/s41598-024-52872-0

**Published:** 2024-04-30

**Authors:** Akbar Mokhtariazar, Amir Hessam Hassani, Mahdi Borghei, Mohamadreza Massoudinejad

**Affiliations:** 1 Department of Environmental Engineering, Islamic Azad University, West Tehran Branch, Tehran, Iran; 2 Department of Environmental Engineering, Faculty of Natural Resources and Environment, Science and Research Branch, Islamic Azad University, Tehran, Iran; 3 Department of Biochemical Engineering and Environmental Control Research Center, Sharif University of Technology, Tehran, Iran; 4 Department of Environmental Health Engineering, School of Public Health and Safety, Shahid Behashti University of Medical Sciences, Tehran, Iran

**Keywords:** Biological techniques, Environmental sciences

## Abstract

Here, a comparative study was designed to survey the treatment efficiency of pharmaceutical wastewater containing Naproxen by Membrane bioreactor (MBR) and MBR with fixed-bed packing media (FBMBR). To this end, the performance of MBR and FBMBR in different aeration conditions including average DO (1.9–3.8 mg/L), different organic loading (OLR) (0.86, 1.14 and 1.92 kg COD per cubic meter per day), and Naproxen removal efficiency. The BOD_5_ removal efficiency, effluent quality and membrane fouling were monitored within 140 days. The results obtained from the present study indicated that COD removal efficiency for FBMBR (96.46%) was higher than that for MBR (95.33%). In addition, a high COD removal efficiency was experienced in both MBR and FBMBR in operational conditions 3 and 4, even where OLR increased from 1.14 to 1.92 kgCOD/m^3^ d and DO decreased from 4 to < 1 mg/L. Furthermore, the higher Naproxen removal efficiency was observed in FBMBR (94.17%) compared to that for MBR (92.76%). Therefore, FBMBR is a feasible and promising method for efficient treatment of pharmaceuptical wastewater with high concentrations of emerging contaminant, especially, the Naproxen.

## Introduction

Environmental pollution is widely known as a major challenge attributed to today's civilization^1^. The industrial and pharmaceutical solid waste, hazardous waste and effluent discharges are the leading causes and agents of water pollution all over the world^2^. Pharmaceutical wastewater as consequences of a variety of production stages including conversion of natural substances into pharmaceutical ingredients through fermentation and extraction are generally characterized with toxic and refractory compounds^3^; the improper discharge of these compounds into environment exacerbate the environmental pollution^4^. It is projected that average daily wastewater generation for pharmaceutical manufacturing unit in U.S.A is 1.0068 × 10^9^ L. Therefore, huge volume of wastewater with complex and hazardous nature is a serious threat for environmental matrices, specially, the groundwater^5^. U.S. E.P.A classify the pharmaceutical wastewater as “red category”, due to high chemical oxygen demand (COD), 5-day biological oxygen demand (BOD_5_), solids contents, supplementary chemicals and presence of pharmaceuticals, antibiotics or their secondary metabolites^6,7^. On the other hand, the conventional wastewater treatment methods are not able to efficiently remove or degrade such refractory and toxic compounds present in pharmaceutical wastewater^8^. In addition, the increased awareness on potential threats of pharmaceutical wastewater make researchers to employ different treatment technologies including activated carbon filter and coagulation, biological treatments, advanced oxidation processes and combined advanced oxidation processes (AOPs) and biological treatment^2,9^ in order to meet standards recommended for discharge into environment. For instance, Changotra et al. (2019) investigated the treatment of pharmaceutical wastewater using combined approach of Fenton applications and aerobic biological treatment^10^. The authors reported that the combined Fenton and aerobic biological technology revealed the complete detoxification of pharmaceutical wastewater^10^. However, the biological process is known as the least expensive and environmental friendly approach in order to treat the wastewater. On the other hand, the modification and improvement on biological treatment process are required to facilitate the wastewater containing the non-biodegradable compounds^11^. Membrane bioreactor (MBR) has recently received much attention as one of the promising technologies for wastewater treatment and reuse^12^. MBRs are able to provide an effluent with higher quality to comply with strict regulation limits^13^. Removal of organic matter, various micronutrients and drugs are one of the most important concerns of pharmaceutical WWTP, which can be treated by the MBR. One of the advantages of MBR over the conventional processes is that the high concentration of microorganisms (above 20,000 mg/L) are incubated in smaller process unit^14^. MBR ensures high-quality effluent, has capability in resisting high organic loading, generates largely disinfected effluent, and limits sludge generation^15^. Raghavan et al. (2017) surveyed on removal behavior of 12 antibiotics using osmotic membrane bioreactor. The authors reported that the high removal efficiency for all antibiotic were 77.7–99.8%^16^. In addition, Zhicheng Xu et al. (2019) reported that sequencing-batch membrane bioreactor led to high removal efficiency (90%) for both sulfonamides and tetracyclines. However, the removal efficiency for fluoroquinolones was lower than 70%^17^. Recent studies have shown that addition of powdered activated carbon (PAC) in MBR would reduce membrane fouling and the operation cost decreased. PAC leads to reduced sludge production and on the other hand improve the quality of effluent quality^18^. Naproxen is one of most common antibiotics used in the hospitals. It is a very strong analgesic which used in the treatment of Crohn's disease. Naproxen has the highest volume of production compared to other drugs in the country, Naproxen sodium, diclofenac potassium and etodolac have both COX-1 and COX-2 inhibitory effects^19^. Naproxen belongs to the category of anti-inflammatory drugs. In general, Naproxen has biological interaction, high absorption and hepatic metabolism, with a half-life of 12–24 h; it is excreted through the gall bladder and a small amount through urine and feces. Some studies have focused on the Naproxen removal efficiency from pharmaceutical wastewater^20–23^. For instance, Chon et al. (2011) reported that the removal efficiency of Naproxen by nano-filtration process were above 78%^24^. In addition, Hashim et al. (2011) surveyed the feasibility of laboratory-scale membrane bioreactor in removal of Naproxen. The authors reported that laboratory-scale membrane bioreactor are able to remove 68%^23^. Therefore, the present study was developed to evaluate a fixed bed with an attached growth medium in MBR in order to evaluate (1) the removal efficiency of Naproxen, (2) removal efficiency of organic pollution and (3) the effects of organic load in removal of pollution in real pharmaceutical wastewater.

## Materials and methods

The present experimental-laboratory study was conducted on a pilot scale fed with real pharmaceutical wastewater. The ability of continuous-mode MBR to reduce organic matter, nutrients and Naproxen from wastewater, and membrane clogging in four operational conditions with different organic loading rates were analyzed in a period of 140 days. In addition, these four operational conditions were compared to evaluate the system's performance in removal of organic matter, nutrients and Naproxen in high and low aeration conditions.

### Wastewater sampling and characterization

The physico-chemical characteristics of the four real pharmaceutical wastewater samples used during the study period are summarized in Table 1.

**Table 1:** Physico-chemical characteristics of raw pharmaceutical wastewater used in the pilot study period.

Parameter	Mean	Min.	Max.
BOD_5_ (mg/L)	397	220	595
COD (mg/L)	608	442	875
PO_4_ (mg/L)	11.5	5.8	14.6
NO_3_ (mg/L)	115	67	149
Naproxen (µg/L)	24,750	24,150	25,640

### Pilot configuration

**Figure 1:**
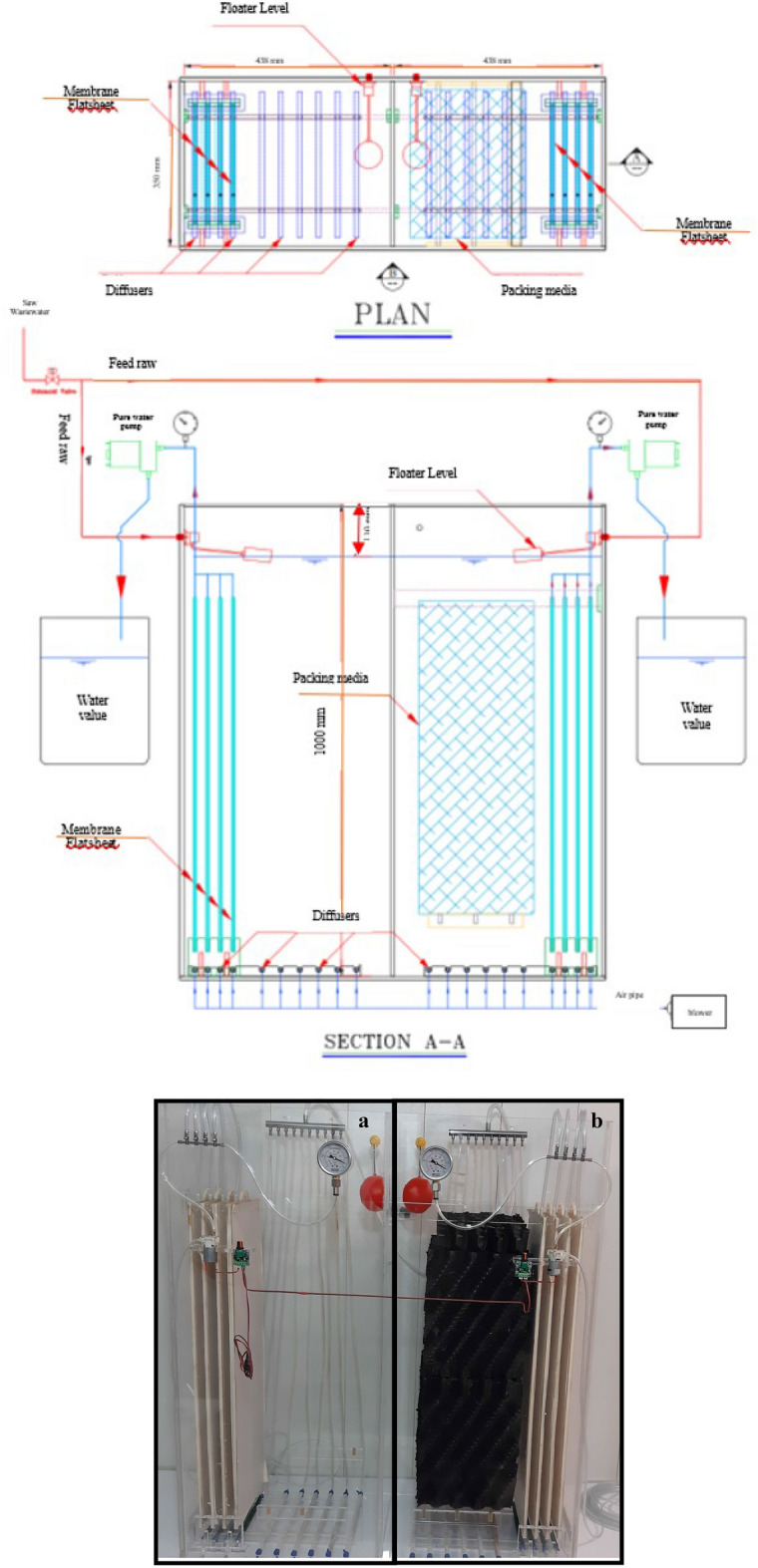
Schematic diagram of MBR without media (**a**) and MBR with packing media (**b**).

The schematic plan of MBR pilot used in the present study is presented in Fig. 1. The rectangular pilot was made of Plexiglas with a thickness of 10 mm. The useful volume of pilot was 140 L (40 cm in length, 35 cm in width and 100 cm in height).

In this study, the raw wastewater collected from one of the pharmaceutical companies was first entered into the feed tank with a volume of 500 L along a day and then entered the pilot with the command of a solenoid valve. An exhaust effluent suction pump with a maximum capacity of 30 L per hour was also installed. In these tanks, pressure gauges were designed to measure the output pressure of the membrane. The bottom of the pilots were equipped with blowers and diffusers for subsurface aeration. In addition, the schematic diagram of MBR without packing media and control are presented in Fig. 1a. Figure 1b illustrates the pilot equipped with membrane and packing media (FBMBR) in order to compare removal efficiency of pollutant with control. The pilot was operated during the six months of April to September at a sewage temperature of about 25 °C. The pilot was gradually fed with 25, 50 and 100%. After 2 months of launching the pilot, the operational parameters including removal of organic matter were analyzed according to the defined scenarios.

### Membrane structure

Technical specifications of polypropylene hollow fiber membrane used in the MBR and present study are summarized in Table 2.

**Table 2:** Technical specifications of polypropylene membrane used in the MBR.

Filter material	Polypropylene	Filter operating temperature	°C
External shape and type	Halo fiber	pH filter performance	0–14
Inner diameter of each fiber	320–350 µm	Size a*c*b	800 × 400 × 50 mm
The external diameter of each fiber	400–450 µm	Membrane area	0.8 m^2^
Pore size	0.1–0.2 µm	Membrane working pressure (suction)	0.1–0.3 Bar
Pore density	40–50%	Filter outlet flow rate	50–80 L h^−1^
X stress resistance	MPa	Number	4
Specific area	350 m^2^/m^3^		

**Figure 2:**
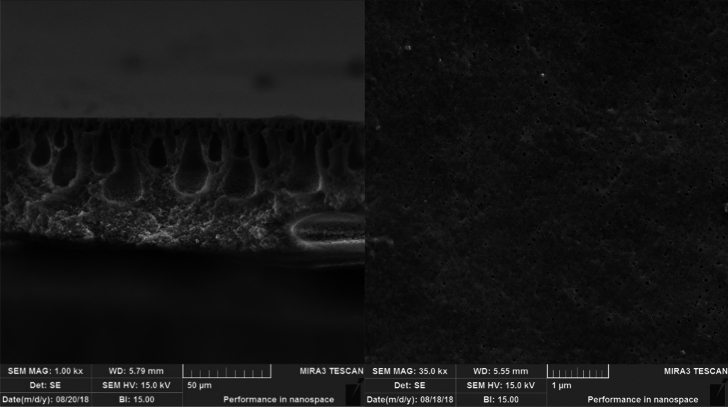
SEM image of membranes used in this study.

Figure 2 shows the surface morphological characteristics of membrane used in the present study. As shown in Fig. 2, the ultra-thin membrane is made of flat sheet using 12% polyvinylidene fluoride solution (PVDF) and has a porous structure with finger porosity with pore diameter between 2 and 8 µm. In addition, the operating pressure of this membrane was between 0.1 and 0.5 bar and the test period stops when it reaches 90% of the maximum pressure. The total area of both sides of each membrane sheet was 0.35 square meters (70 cm long and 25 cm wide).

### MBR operational performances

In this study, the performance of MBR in different aeration conditions, average concentration of dissolved oxygen equal to 3.8 mg/L and 1.9 mg/L, and different organic loading (OLR) on COD, Naproxen removal efficiency were investigated. In three cases, the average of 0.86, 1.14 and 1.92 kg COD per cubic meter per day was fed into the MBR reactors. The detailed information on different operational conditions were summarized in Table 3. The average organic load of the reactor was the average of the values measured during the testing and pilot studies, and therefore the organic load varies around the mentioned numbers according to the input and operating conditions.

**Table 3:** Operational conditions of two pilots.

Period (days)	Scenario	HRT (h)	OLR (kgCOD/m^3^d)	DO (mg/L)	MLVSS (mg/L)	
					MBR	FBMBR
1–60	Start-up	0	0.86	> 4	1730	1980
61–90	S1	48	1.14	> 4	2230	2440
91–120	S2	24	1.14	> 4	2790	3120
121–150	S3	12	1.92	< 1	3185	3090
151–180	S4	6	1.92	< 1	3255	3445

### Analytical analysis

Sampling of raw wastewater and effluent from the membrane bioreactor was performed weekly by measuring the parameters of BOD_5_, COD, total nitrogen (TN), total phosphorous (TP) and naproxen from the pilot inlet and outlet and were analyzed based on procedures outlined in Table 4 and standard methods for water and wastewater examination^25^. Spectrophotometer (HACH) model DR5000 was used to measure the COD parameter and nutrient composition of wastewater and BOD meter model BODTrakTM was used to measure the BOD_5_ parameter. In addition, each exploitation scenario was monitored over a period of 30 days. Moreover, during the sampling periods, membrane pressure and plugging around the membrane (TMP) were analyzed to identify possible membrane blockage based on previous studies. The residual concentration of Naproxen in different samples were determined by a high-performance liquid chromatography (HPLC, Cecil CE4100) with a UV detector (CE 4900) at 270 nm. A discovery C18 column (250 × 4.6 mm) was employed to measure residual Naproxene concentration, and the analyses were carried out with mobile phase of 42% acetonitrile and 58% water adjusted to pH 3 using phosphoric acid. Table 4 summarizes the specifications of the test method and test tools.

**Table 4:** Standard test method for qualitative parameters.

Parameter	Devices	Procedure
BOD5	BODTrakTM	APHA (2005)-5210 B
COD	HACH DR5000	APHA (2005)-5220 D
NO3	HACH DR5000	APHA (2005)-4500 NO3
PO4	HACH DR5000	APHA (2005)-4500 P
Naproxen	Liquid HPLC	APHA (2005)-6810

## Results and discussion

### Operational performance of MBR and FBMBR

#### COD removal efficiency

**Figure 3:**
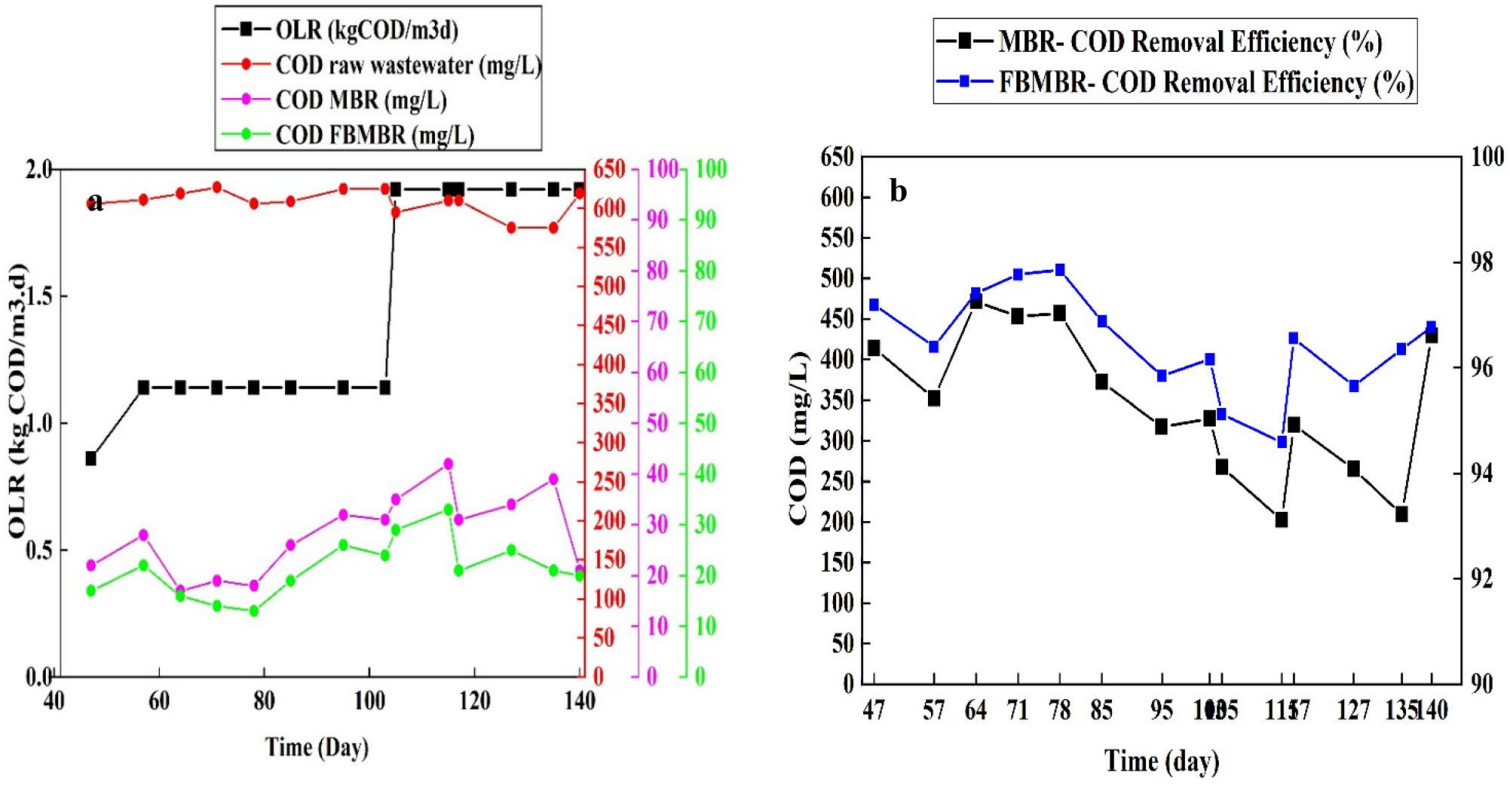
Trends of COD (**a**) and COD removal efficiency (**b**) in MBR and FBMBR for different scenario and OLR.

The variation of COD and removal efficiency in MBR and FBMBR for different scenarios and OLR within the study period is shown in Fig. 3a–b. As illustrated in Fig. 3a, the COD for raw wastewater during the study period were between 575 and 625 mg/L; there is a little variation in wastewater fed into both MBR and FBMBR. In addition, as illustrated in Fig. 3a, the average COD for samples withdrawn from MBR (min: 17, max: 42, average: 28.21 mg/L) were higher those taken from FBMBR (min: 13, max: 33, average: 21.42 mg/L) within operation time. Moreover, the trends of COD removal efficiency for MBR and FBMBR are illustrated in Fig. 3b. As shown in Fig. 3b, the average COD removal efficiency for FBMBR (96.46%) was higher than that for MBR (95.33%). Furthermore, a high COD removal efficiency was observed for both MBR and FBMBR in operational conditions 3 and 4, where OLR increased from 1.14 to 1.92 kgCOD/m^3^ d and DO decreased from 4 to < 1 mg/L. Kaya et al. (2016) surveyed the pharmaceutical wastewater treatment using MBR equipped by powdered activated carbon (PAC)^26^. The results indicated that PAC improved the COD removal efficiency by 10%; the COD removal efficiency in MBR without PAC was 81%^27^.One possible reason for higher COD removal efficiency in FBMBR is related to higher growth of biofilm on packing media^28^. In addition, the ANOVA statistical analysis showed that there is no significant differences between the COD removal efficiency in MBR for different OLR and operational conditions (*P*-value < 0.05); the higher (97.25%) and lower (93.22%) COD removal efficiency were observed in OLR equal to 1.14 kgCOD/m^3^ d and 1.92 kgCOD/m^3^ d, respectively. While, as for FBMBR, a significant differences was observed in COD removal efficiency for different OLR and operational conditions (*P*-value > 0.08).

#### BOD_5_ removal efficiency

**Figure 4:**
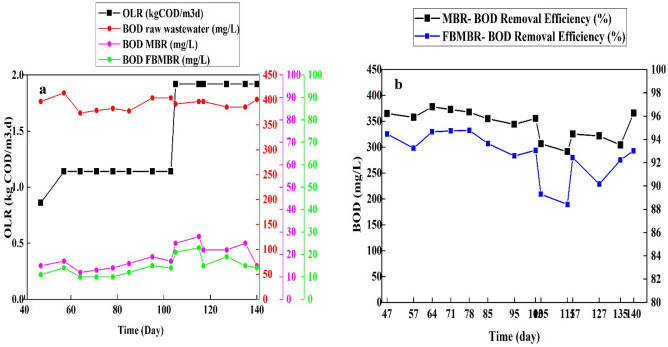
Trends of BOD_5_ (**a**) and BOD_5_ removal efficiency (**b**) in MBR and FBMBR for different scenario and OLR.

The variation of BOD_5_ and removal efficiency in MBR and FBMBR for different operational parameters and OLR within the study period is shown in Fig. 4a–b. As illustrated in Fig. 4a, the BOD_5_ for raw wastewater during the study period were between 378 and 417 mg/L; there is a little variation in BOD of wastewater fed into both MBR and FBMBR. As illustrated in Fig. 4a, the average BOD_5_ of samples withdrawn from FBMBR (min: 10, max: 23, average: 14.5 mg/L) were lower than that for MBR (min: 12, max: 28, average: 18.57 mg/L). In addition, the trends of BOD_5_ removal efficiency for MBR and FBMBR are also illustrated in Fig. 4b. As shown in Fig. 4b, the average BOD_5_ removal efficiency for FBMBR (96.31%) was higher than that for MBR (95.27%). Furthermore, the higher and lower BOD_5_ removal efficiency were attributed to OLR equal to 1.14 kgCOD/m^3^ d to 1.92 kgCOD/m^3^ d, respectively. The ANOVA statistical analysis indicated that there are significant differences between the BOD_5_ removal efficiency by variation of OLR as an influencing parameter in MBR (*p*-value = 0.007) and FBMBR (*p*-value = 0.009). As earlier mentioned, the most possible reason for higher BOD_5_ removal in FBMBR is attributed to higher growth of biofilm on packing media^28^.

#### Naproxen removal efficiency

**Figure 5:**
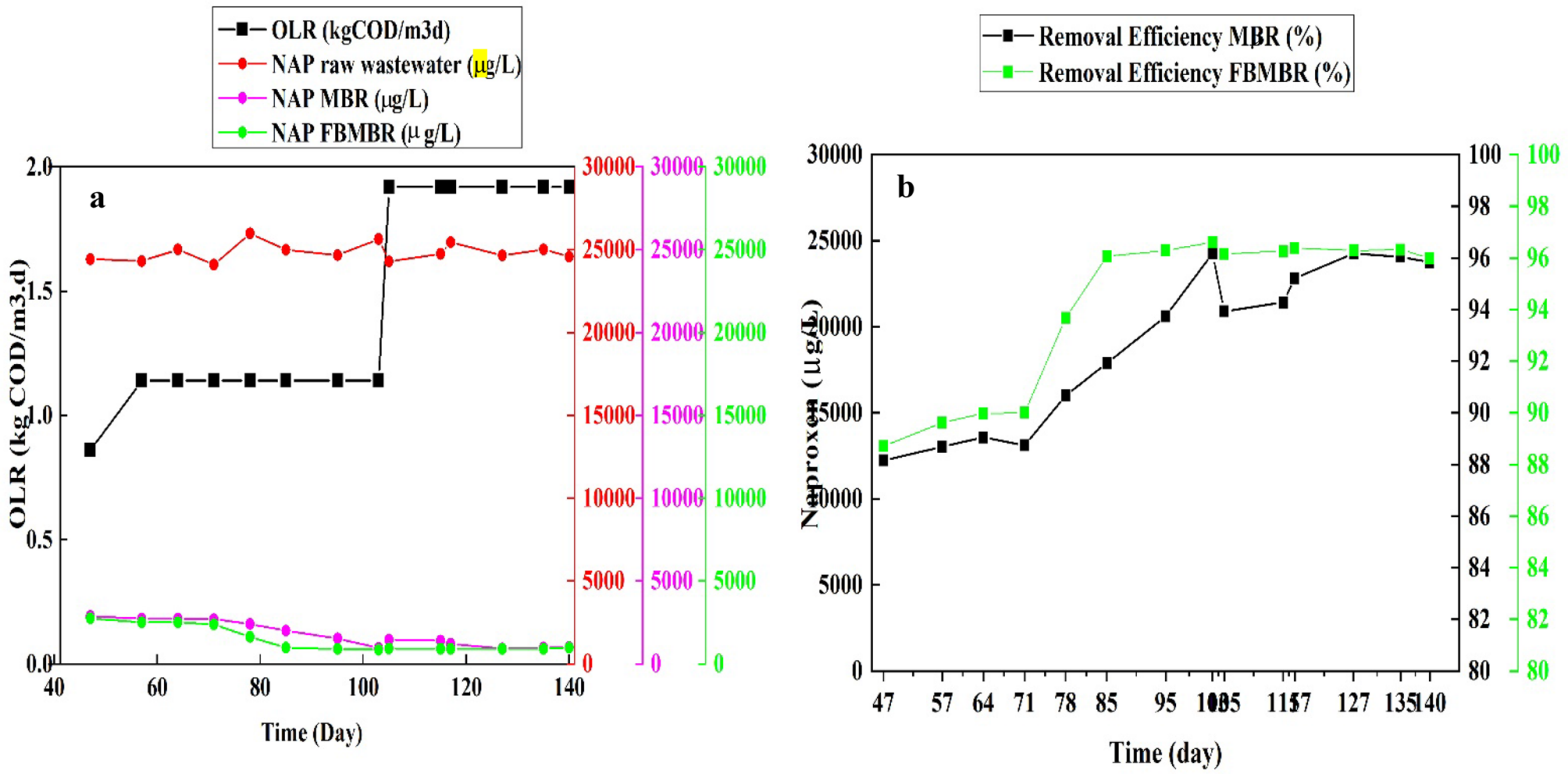
Trends of NAP (**a**) and NAP removal efficiency (**b**) in MBR and FBMBR for different scenario and OLR.

Figure 5a–b illustrate the trend of Naproxen concentration and Naproxen removal efficiency during the study period as a factor of OLR in different scenarios. As shown in Fig. 5a, the initial concentration of Naproxen in raw wastewater were within 24,105–25,985 µg/L. In addition, the average Naproxen concentration in effluent samples withdrawn from FBMBR (min: 870, max: 2760, average: 1444.28 µg/L) were lower than that for MBR (min: 945, max: 2895, average: 1795.71 mg/L). In addition, the trends of Naproxen removal efficiency for MBR and FBMBR are also illustrated in Fig. 5b. As shown in Fig. 5b, the average Naproxen removal efficiency for FBMBR (94.17%) was higher than that for MBR (92.76%). Chon et al. (2011) reported that nano-filtration is able to remove over 78% of Naproxene^24^, while Radjenovic et al. (2007) observed the efficient removal efficiency (99%) of naproxen when two flat-sheet mode membrane with HRT equal to 14 h was employed^29^. In addition, Gurung et al. (2019) studied the removal and fate of emerging organic micropollutants (EOMs) in municipal wastewater by a pilot-scale membrane bioreactor (MBR) treatment and the results indicated that naproxen removal efficiency was between 96 and 99.9%^21^, which are comparable with our results. The ANOVA statistical analysis indicated that there are significant differences between the Naproxen removal efficiency by variation of OLR as an influencing parameter in MBR (*p*-value = 0.009) and FBMBR (*p*-value = 0.023); the highest and lowest Naproxen removal efficiency were observed in OLR of 1.92 and 0.86 kg COD/m^3^ d), respectively.

#### Nutrient removal efficiency

Nitrogen (N) and phosphorus (P) are the most common chemical parameters of effluent before discharge into environment. N and P are the leading causes of eutrophication in the water bodies. Therefore, it is necessary to develop the applicable technologies for simultaneous biological removal of N and P from wastewater to be treated^30^. The results indicated that the average NO_3_ and PO_4_^3−^ removal efficiency in MBR were 89.32% and 60.49%, respectively. While, the removal efficiency of NO_3_ and PO_4_^3−^ in FBMBR were 89.84% and 62.02%, respectively. Overall, the removal efficiency of NO_3_ and PO_4_^3−^ in FBMBR were higher than those in MBR. Li et al. (2023) studied on nitrogen and phosphorous removal efficiency via MBR reactors. The authors reported that MBR can remove the total nitrogen and total phosphorus until 90.1% and 57.8%, respectively, which are similar to results obtained from the present study^31^.

### Membrane fouling

**Figure 6:**
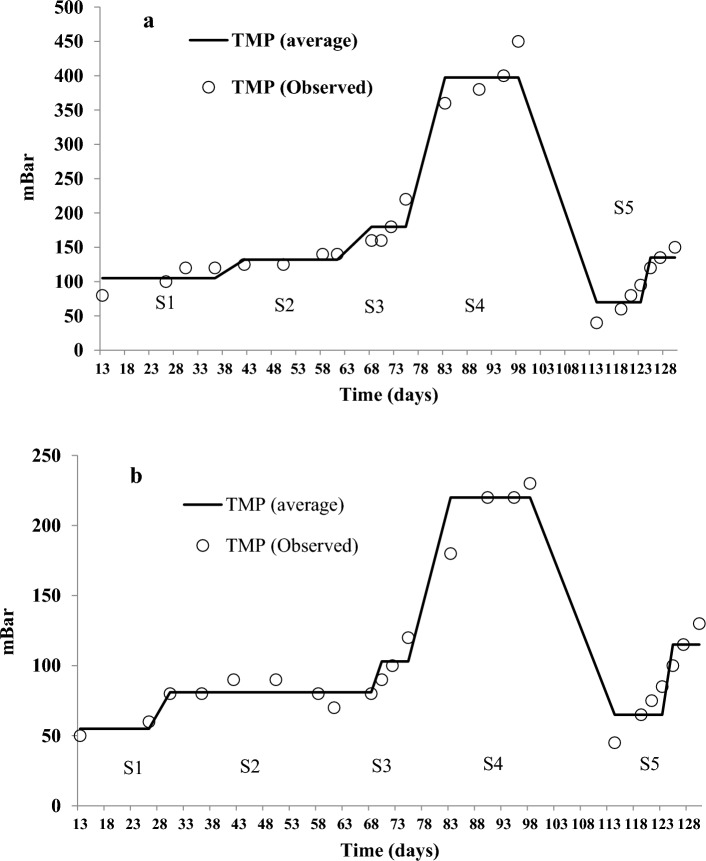
TMP in MBR (**a**) and FBMBR (**b**) for different scenario and OLR.

The transmembrane pressure (TMP) of MBR and FBMBR pilots as function of OLR during the operational period are illustrated in Fig. 6a–b. As illustrated in Fig. 6a–b, the head drop of the flow passing through the membrane increases over time with increasing sludge age. In addition, increased the OLR consequently decrease the hydraulic retention time, and reducing the amount of aeration are also effective in increasing the severity of membrane clogging. Of note, the similarity of head drop in both systems indicates that the development of the hybrid system does not change the way of membrane biofilm formation and clogging; the operating conditions of these systems, such as the amount of aeration, sludge age, and the amount of organic loading, are still effective factors on the amount of clogging. The main reasons for membrane fouling are attributed to deposition and adsorption of solutes and colloidal particles (internal fouling) or deposition of particles, colloids and macromolecules on the membrane surface is called external fouling^32^.

## Conclusion

The present study was developed to investigate the removal efficiency of Naproxen in pharmaceutical using of MBR and FBMBR within incubation period of 140 days and different scenarios. In this study, BOD_5_, COD, Naproxen removal efficiencies, effluent quality and membrane fouling were surveyed. The results indicated that fixed-bed packing media in FBMBR promote the Naproxene removal efficiency; Naproxene (94.17%) removal efficiency were observed in FBMBR even where even where OLR increased from 1.14 to 1.92 kgCOD/m^3^ d and DO decreased from 4 to < 1 mg/L. The present study showed that FBMBR with packing media is a promising approach to treat the pharmaceutical wastewater with high concentration of antibiotics and emerging contaminants.

## Data Availability

All data generated or analysed during this study are included in this published article.

## References

[1] Rana RS (2017). A review on characterization and bioremediation of pharmaceutical industries’ wastewater: An Indian perspective. Appl. water Sci..

[2] Moghaddam A (2023). Biodegradation of pharmaceutical compounds in industrial wastewater using biological treatment: A comprehensive overview. Int. J. Environ. Sci. Technol..

[3] Mascolo G (2010). Biodegradability of pharmaceutical industrial wastewater and formation of recalcitrant organic compounds during aerobic biological treatment. Bioresour. Technol..

[4] Cunningham, V. L. *et al.* Effects of human pharmaceuticals on aquatic life: next steps (2006). 10.1021/es063017b 16786680

[5] Adishkumar S, Sivajothi S, Rajesh Banu J (2012). Coupled solar photo-fenton process with aerobic sequential batch reactor for treatment of pharmaceutical wastewater. Desalin. Water Treat..

[6] Gadipelly C (2014). Pharmaceutical industry wastewater: Review of the technologies for water treatment and reuse. Ind. Eng. Chem. Res..

[7] Martínez F (2018). Techno-economical assessment of coupling Fenton/biological processes for the treatment of a pharmaceutical wastewater. J. Environ. Chem. Eng..

[8] Lefebvre O, Shi X, Wu CH, Ng HY (2014). Biological treatment of pharmaceutical wastewater from the antibiotics industry. Water Sci. Technol..

[9] Liu L (2021). Treatment of industrial dye wastewater and pharmaceutical residue wastewater by advanced oxidation processes and its combination with nanocatalysts: A review. J. Water Process Eng..

[10] Changotra R, Rajput H, Dhir A (2019). Treatment of real pharmaceutical wastewater using combined approach of Fenton applications and aerobic biological treatment. J. Photochem. Photobiol. A Chem..

[11] Carballa M (2004). Behavior of pharmaceuticals, cosmetics and hormones in a sewage treatment plant. Water Res..

[12] Rahman TU (2023). The advancement in membrane bioreactor (MBR) technology toward sustainable industrial wastewater management. Membranes (Basel).

[13] Mannina G (2021). Integrated membrane bioreactors modelling: A review on new comprehensive modelling framework. Bioresour. Technol..

[14] Iorhemen OT, Hamza RA, Tay JH (2016). Membrane bioreactor (MBR) technology for wastewater treatment and reclamation: membrane fouling. Membranes (Basel).

[15] Deng L (2020). Application of a specific membrane fouling control enhancer in membrane bioreactor for real municipal wastewater treatment: Sludge characteristics and microbial community. Bioresour. Technol..

[16] Raghavan DSS, Qiu G, Ting Y-P (2018). Fate and removal of selected antibiotics in an osmotic membrane bioreactor. Chem. Eng. J..

[17] Zheng W, Wen X, Zhang B, Qiu Y (2019). Selective effect and elimination of antibiotics in membrane bioreactor of urban wastewater treatment plant. Sci. Total Environ..

[18] Pulpeiro, M. G., Pavlović, D. M. & Stipaničev, D. Improvement in the pharmaceutical removal from hospital wastewater in a full-scale hybrid PAC-MBR. In *6th IWA Specialized International Conference on eco-Technologies for Wastewater Treatment (ecoSTP) 2023* (2023).

[19] Akbulut N, Üstüner E, Atakan C, Çölok G (2014). Comparison of the effect of naproxen, etodolac and diclofenac on postoperative sequels following third molar surgery: A randomised, double-blind, crossover study. Med. Oral Patol. Oral Cir. Bucal.

[20] Kwon Y, Lee DG (2019). Removal of contaminants of emerging concern (CECs) using a membrane bioreactor (MBR): A short review. Glob. Nest J..

[21] Gurung K, Ncibi MC, Sillanpää M (2019). Removal and fate of emerging organic micropollutants (EOMs) in municipal wastewater by a pilot-scale membrane bioreactor (MBR) treatment under varying solid retention times. Sci. Total Environ..

[22] Quintana JB, Weiss S, Reemtsma T (2005). Pathways and metabolites of microbial degradation of selected acidic pharmaceutical and their occurrence in municipal wastewater treated by a membrane bioreactor. Water Res..

[23] Hashim NH, Nghiem LD, Stuetz RM, Khan SJ (2011). Enantiospecific fate of ibuprofen, ketoprofen and naproxen in a laboratory-scale membrane bioreactor. Water Res..

[24] Chon K (2011). Evaluation of a membrane bioreactor and nanofiltration for municipal wastewater reclamation: Trace contaminant control and fouling mitigation. Desalination.

[25] APHA, AWWA, W. Standard Methods for examination of water and wastewater. *American Public Health Association (APHA)* (2017).

[26] Kaya Y (2016). Improving the performance of an aerobic membrane bioreactor (MBR) treating pharmaceutical wastewater with powdered activated carbon (PAC) addition. Bioprocess Biosyst. Eng..

[27] Sanderson H (2004). Ranking and prioritization of environmental risks of pharmaceuticals in surface waters. Regul. Toxicol. Pharmacol..

[28] Arya V, Philip L, Bhallamudi SM (2016). Performance of suspended and attached growth bioreactors for the removal of cationic and anionic pharmaceuticals. Chem. Eng. J..

[29] Radjenovic J, Petrovic M, Barceló D (2007). Analysis of pharmaceuticals in wastewater and removal using a membrane bioreactor. Anal. Bioanal. Chem..

[30] Zhou Q, Sun H, Jia L, Wu W, Wang J (2022). Simultaneous biological removal of nitrogen and phosphorus from secondary effluent of wastewater treatment plants by advanced treatment: A review. Chemosphere.

[31] Li C, Du X, Huang C, Zhang Z (2023). Effects of high pharmaceutical concentrations in domestic wastewater on membrane bioreactor treatment systems: Performance and microbial community. Membranes (Basel).

[32] Du X, Shi Y, Jegatheesan V, Haq IU (2020). A review on the mechanism, impacts and control methods of membrane fouling in MBR system. Membranes (Basel).

